# Baseline Peripheral Blood Leukocytosis Is Negatively Correlated With T-Cell Infiltration Predicting Worse Outcome in Colorectal Cancers

**DOI:** 10.3389/fimmu.2018.02354

**Published:** 2018-10-12

**Authors:** Xiang Hu, Ya-Qi Li, Qing-Guo Li, Yan-Lei Ma, Jun-Jie Peng, San-Jun Cai

**Affiliations:** ^1^Department of Colorectal Surgery, Fudan University Shanghai Cancer Center, Shanghai, China; ^2^Department of Oncology, Shanghai Medical College, Fudan University, Shanghai, China

**Keywords:** leukocytosis, colorectal cancer, prognostic factor biomarker, immune microenvironment, tumor-infiltrating-lymphocytes

## Abstract

We aimed to explore the prognostic value of blood leukocyte and to generate a predictive model to refine risk stratification for colorectal cancers. 6,558 patients with colorectal cancers were identified eligible respectively in Fudan University Shanghai Cancer Center (FUSCC) between May, 2008 and October, 2016. Then the entire set is divided into a training set and a testing set. The prognostic value of pretreatment white blood cell count and clinicopathologic parameters in the context of tumor-infiltrating lymphocytes (TIL) and neutrophils was investigated. Conventional leukocytosis (≥10,000/μl) was significantly associated with decreased overall survival (OS) and disease-free survival (DFS) (*p* < 0.05). In fact, moderately elevated leukocyte (≥7,500/μl) has also been identified as an independent prognostic factor for survivals in the training, testing, and entire sets, respectively. And leukocytosis correlated with advanced T-stage (*p* < 0.001), M-stage (*p* < 0.001), poor differentiation tumor (*p* = 0.023) and Glasgow prognostic score, even predicted for worse relapse postoperatively (*p* = 0.001) and resistance to chemotherapy. In addition, nomograms on OS and DFS were established according to leukocytosis and other significant factors, demonstrating a great prediction accuracy. Importantly, pretreatment leukocytosis had a significantly lower intra-tumor CD3+ and CD8+ TIL infiltration (*p* < 0.001 and *p* = 0.033), whereas low CD3+ and CD8+ TIL expression in tumor were associated with worse OS and DFS (*p* = 0.02 and *p* = 0.015). In conclusion, our study validates leukocytosis as an independent prognostic factor in colorectal cancers. Our data provide for the first-time vital insight on the correlation of peripheral pretreatment leukocytosis with the tumor-infiltrating cells contexture and might be relevant for future risk stratification.

## Introduction

Growing evidences have emerged in recent years that inflammation may be the origin of many malignancies (1, 2). As the third most commonly diagnosed malignant tumor worldwide, colorectal cancer (CRC) represents a growing number of cancers associated with inflammation (3). Surgical resection and peri-operative multimodal treatment remain the hope for curative treatment of colorectal cancer. However, the mortality-to-prevalence ratio (MPR) of CRC in China remains 14%, indicating that survival outcome of cancer in China is apparently worse (4). A high proportion of CRC still resists to the chemotherapy and eventually develops relapse within 2 years post-operation. Thus, a concise model to predict the efficacy of chemotherapy and prognosis of CRC patients is urgently needed. Although the current prognostic model applied in clinical practice for risk stratification and treatment management mainly depends on tumor cell-centered stratification systems (TNM staging system), but even patients with the same stage can have very distinct prognosis (5). As this staging system narrowly concentrated on the tumor cells without incorporating the effects of the host inflammation and immune response. Being an inflammatory-associated tumor, colorectal cancer is characterized by infiltration of heterogeneous immune cells and peripheral hematologic profile disorder, which configure the complicated microenvironment affecting tumor development (6). Prognostic serum marker such as, leukocyte counts, is easier to use in bedside, which are our focus and under investigation. In oropharyngeal cancer, anal carcinoma and cervical cancer, the worse outcome of elevated pretreatment white blood cell (WBC) count in peripheral blood has already been indicated (7–9). However, no study so far considered WBC to evaluate its prognostic value in colorectal cancer, and the optimal cut-off value for leukocytosis to predict prognosis was not determined. In addition, no explanation for this phenomenon has been provided so far. Hence, we aim to assess the association of pretreatment white blood cell to patient clinical and molecular characteristics, as well as prognosis. Furthermore, we will provide insight on the clinical outcome of leukocytosis in correlation with microenvironment context.

## Materials and methods

### Study population

A retrospective analysis was performed in patients with colorectal cancers from Fudan University Shanghai Cancer Center (FUSCC) between May, 2008 and October, 2016. At the meantime, a training set and a testing set, comprising consecutive patients in the year of 2008-2012 and patients in the year of 2013-2016, were obtained from the entire cohort. Only patients with fully characterized tumors, intact overall survival (OS) and disease-free survival (DFS) information were included. All patient data are retrieved, including age, gender, diagnostic year, Albumin, C-reactive protein (CRP) tumor location, primary tumor size, histological grade, number of lymph nodes examined, type of chemotherapy, details of the surgical procedure, occurrence of complications, postoperative staging, and follow-up (date of last visit, tumor local recurrence, distant metastasis, overall and disease-free survival). The exclusion criteria were as follows: previously applying anti-inflammatory medicines or immunosuppressive therapy received, such as recent steroid exposure, or with chronic inflammatory diseases including autoimmune diseases and infections; neoadjuvant therapy received. In all, 6,558 patients were enrolled in this study with the entire cohort. Then a training set comprising 3,798 patients and a testing set with 2,760 patients were obtained. All patients from FUSCC dataset have provided written informed consent. The research protocol was reviewed and approved by the institutional review board of the FUSCC.

### Immunohistochemistry (IHC)

Immunohistochemical staining of CD3, CD8, CD45, CEACAM8 was performed by a horseradish-peroxidase technique with appropriate antibodies (anti-CEACAM8 antibody, BD Biosciences; anti-CD3e, CD8a, CD45 from Cell Signaling Technology). The number of positive cells per field was estimated using Image Pro plus 6.0 (Media Cybernetics Inc., Bethesda, MD). The immunostaining was evaluated by two pathologists blinded to the clinical information (10). The cutoff values for survival analysis was determined by X-tile 3.6.1 software (11) (Yale University, New Haven, CT, USA).

### Peripheral blood counts

We derived the pretreatment baseline leukocyte counts from our hospital database. Baseline was defined as before the start of surgery employed in the current analysis.

### Statistical analysis

Statistical evaluation was performed using IBM SPSS statistics Version 22 (SPSS Inc; IBM Corporation Software Group, Somers, NY). The Chi-square test or Fisher exact test was utilized in exploratorily comparing baseline characteristics. For pairwise analysis in continuous variables, the correlation with blood leukocyte and tumor infiltration cells was calculated by spearman. OS and DFS were compared with the Kaplan-Meier plotter and the log-rank (Mantel-Cox) test to identify the independent survival beneficial subgroups. Uni- and multivariate analysis of prognostic factors for survival were conducted by cox proportional hazard models to investigate the effect on survival among covariates including the diagnostic age, sex, disease stage, histological type, differential grade and adjuvant treatment. Hazard ratios (HRs) and 95% confidence intervals (CIs) for multivariate analyses were computed using the Cox proportional hazards regression models. Receiver operating characteristics (ROC) analyses were performed to identify the performance of blood leukocytosis. The R software version 3.4.3 and the “rms” package (R Foundation for Statistical Computing) were applied to perform the nomogram analysis and calibration plot. All statistical tests were performed 2-sided, and *P*-values < 0.05 were considered to be statistically significant.

## Results

### Prognostic value of leukocyte disorders

**Figure 1:**
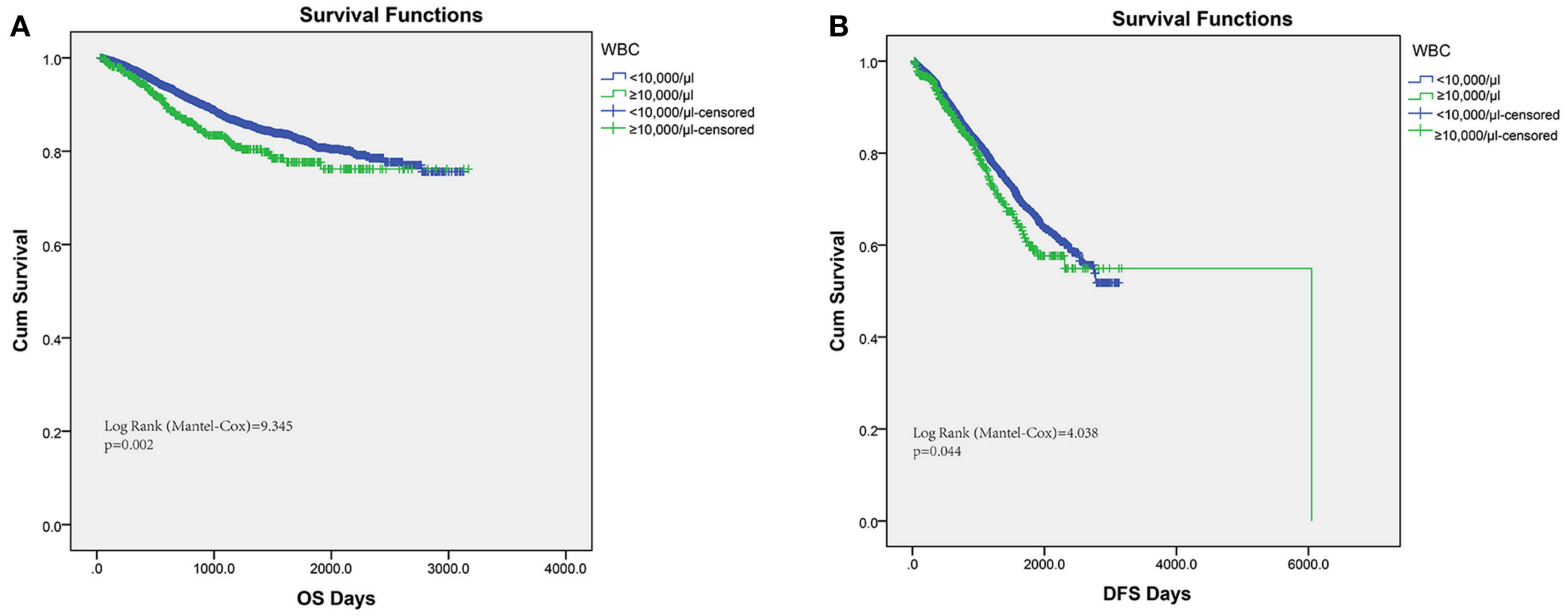
Kaplan–Meier analysis of OS **(A)** and DFS **(B)** based on pretreat leukocytosis defined as leukocyte count over 10,000/μl from the entire set of FUSCC.

Conventionally, pretreatment leukocytosis was defined as the detection of a leukocyte count over 10,000/μl. Hence, among the 6,558 consecutive colorectal cancer patients in this study, pretreatment leukocytosis (leukocyte ≥10,000/μl) was observed in 9.19% (603 out of 6,558). In order to assess the association of pretreatment leukocytosis and cancer prognosis, we performed Kaplan–Meier survival analysis and log-rank tests for the overall survival and disease-free survival. A significantly inferior OS was found in the leukocytosis group (Figure 1A, log-rank test = 9.345, *P* = 0.002) and the consistent association of leukocytosis with regard to DFS was also observed (Figure 1B, log-rank test = 4.038, *P* = 0.044). Furthermore, clinicopathological parameters for predicting OS and DFS were also explored by uni- and multi-variate analysis with Cox regression model. In both models of Cox regression, leukocytosis were consistently validated to be an independent negative prognostic factor for OS and DFS (*p* < 0.05).

### Identification of leukocytosis optimal cut-off values

**Figure 2:**
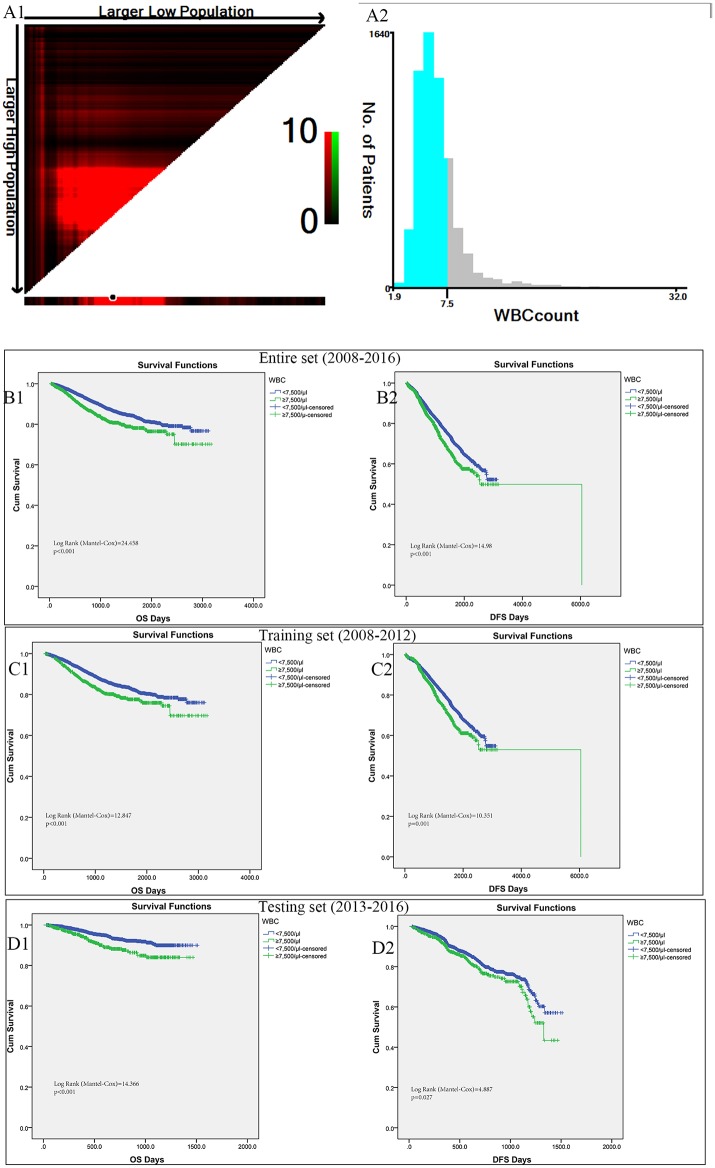
X-tile analyses of OS and DFS were performed to determine the optimal cut-off values for leukocyte count. **(A1,A2)** The optimal cut-off values for leukocyte count from the entire set of FUSCC. Kaplan–Meier analysis of OS **(B1)** and DFS **(B2)** based on pretreat leukocytosis defined as leukocyte count over 7,500/μl from the entire set of FUSCC. **(C1,C2)** Training set comprising 3798 consecutive patients in the year of 2008-2012. **(D1,D2)** Testing set with 2760 consecutive patients in the year of 2013-2016.

In the entire cohort, X-tile program was utilized to identify the optimal cut-off values of leukocyte count for prognosis, which was 7,500/μl (Figures 2A1,A2). Then patients with leukocytosis and non-leukocytosis group were identified for further analysis (<7,500/μl as non-leukocytosis group and ≥7,500/μl as leukocytosis group). Kaplan–Meier survival analysis revealed that leukocytosis was significantly associated with decreased OS and DFS (all *p* < 0.001, Figures 2B1,B2). To guarantee leukocytosis as a universal application across heterogeneous population, the entire cohort has been divided into the training set and testing set comprising 3,798 consecutive patients in the year of 2008–2012 and 2,760 consecutive patients in the year of 2013–2016, respectively. Consistently, patients with leukocytosis displayed significant survival inferiority compared with non-leukocytosis group in the training (Figures 2C1,C2; *P* < 0.001) and testing sets (Figures 2D1,D2; *P* < 0.001).

### Patient and pre-treatment characteristics

**Figure 3:**
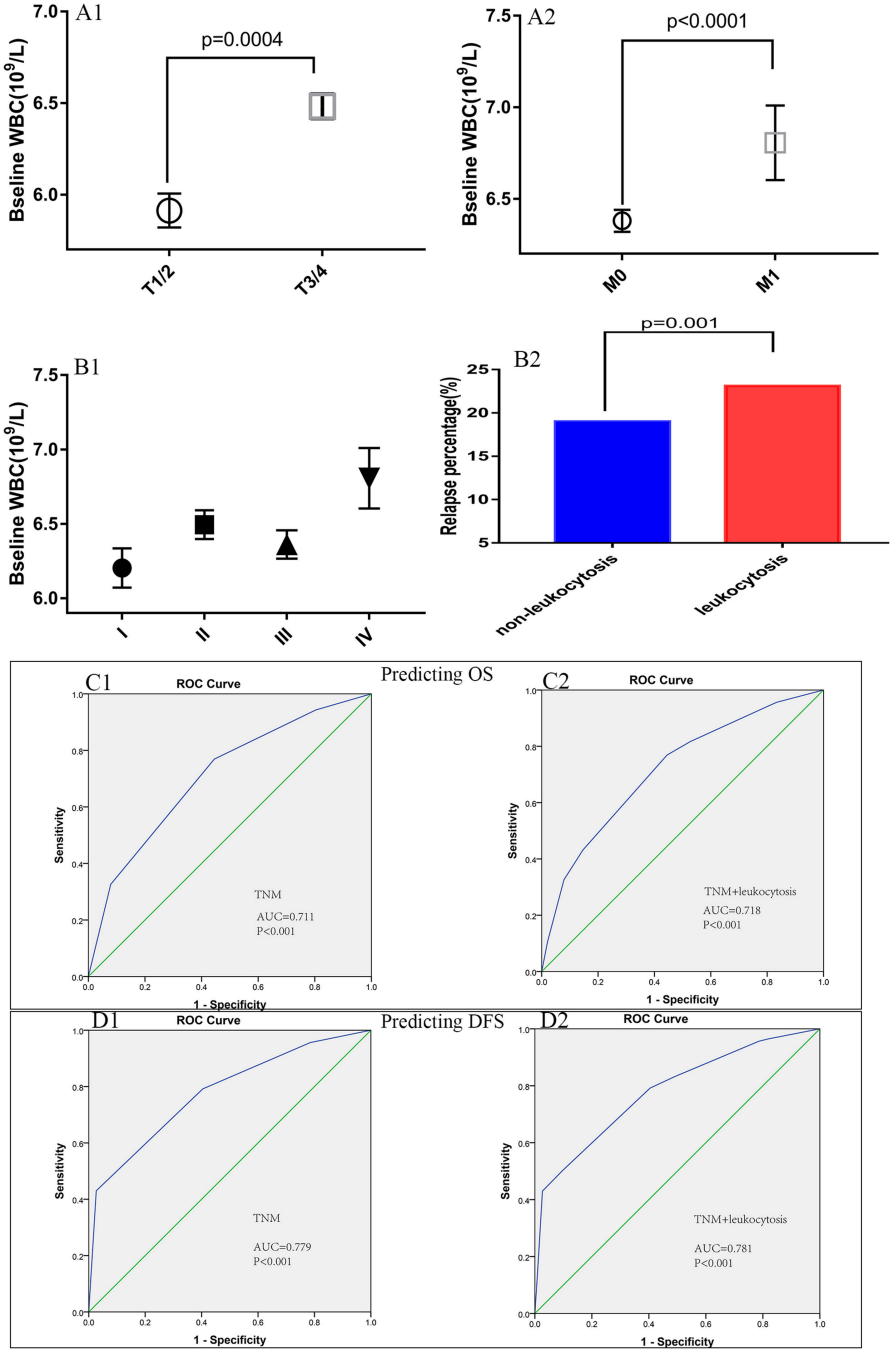
Association of peripheral white blood cell (WBC) with **(A1)** T-stage, **(A2)** M-stage, **(B1)** TNM stage, and **(B2)** relapse rates. The receiver operating characteristic (ROC) curves for predicting OS **(C1,C2)** and DFS **(D1,D2)** using leukocytosis, TNM staging system or a combination of the two factors.

Patient baseline characteristics are summarized in Table 1. Of the entire cohort, 1,369 patients (20.8%) had leukocytosis at baseline, when 7,500/μl was defined as the cutoff value. The median follow-up was 55 months. The median age was 60 years (range 18–96) and 58.8% patients were male. According to 7th AJCC tumor classification, TNM I, II, III, and IV stage distributions of colorectal cases were 1,185 (18.1%), 2,217 (33.8%), 2,456 (37.5%), and 700 (10.7%), respectively. The rate of leukocytosis was significantly more in patients with advanced T stage, M stage, TNM stage and poor differentiation tumor (all *p* < 0.05). At the meantime, advanced T-stage (*p* = 0.0004), M-stage (*p* < 0.0001) and TNM-stage were significantly associated with an elevated WBC count (Figures 3A1,A2,B1). In addition, the Glasgow prognostic score (GPS) based on serum CRP (an acute-phase response protein) and albumin levels (a typical index of malnutrition) was developed to aid in appreciating the role of leukocytosis. In brief, elevated CRP (≥10 mg l^−1^) were allocated an GPS score 1 or 2 depending on the status of hypo-albuminaemia (<35 g l^−1^), whereas normal CRP level (<10 mg l^−1^) are scored 0, even though hypo-albuminaemia is present. It turned out that leukocytosis showed significant relationship with the elevated GPS score.

**Table 1:** Baseline characteristics in the entire cohort based on leukocytes count.

**Variables, N (%)**	Baseline leukocytes (7.5*10^**9**^**/L)**		***P-value***
	**Non-leukocytosis (*n* = 5189)**	**leukocytosis (*n* = 1369)**	
**GENDER**			
Female	2223 (42.8)	475(34.7)	<0.001
Male	2966 (57.2)	894 (65.3)	
Age, years	59.6 ± 12.1	59.3 ± 12.3	0.39
TNM stage			<0.001
I	998 (19.2)	187 (13.7)	
II	1705 (32.9)	512 (37.4)	
III	1985 (38.3)	471 (34.4)	
IV	501 (9.7)	199 (14.5)	
T stage			<0.001
T1	335 (6.5)	56 (4.1)	
T2	924 (17.8)	195 (14.2)	
T3	77 (1.5)	17 (1.2)	
T4	3853 (74.3)	1101 (80.4)	
N stage			0.406
N0	2820 (54.3)	752 (54.9)	
N1	1427 (27.5)	354 (25.9)	
N2	942 (18.2)	263 (19.2)	
M stage			<0.001
M0	4688 (90.3)	1170 (85.5)	
M1	501 (9.7)	199 (14.5)	
Grade			0.023
Well	109 (2.2)	18 (1.4)	
Moderate	3821 (76.1)	982 (74.2)	
Poor	1090 (21.7)	324 (24.5)	
Histological type			0.112
Adenocarcinoma	4836 (93.5)	1256 (92.1)	
Mucinous	292 (5.6)	97 (7.1)	
Lymph node examined			0.03
Median	15 ± 6	16 ± 7	
Perineural invasion			0.412
Negative	4098 (79.6)	1069 (78.5)	
Positive	1053 (20.4)	292 (21.5)	
Vascular invasion			0.724
Negative	3838 (74.0)	1011 (73.8)	
Positive	1293 (24.9)	346 (25.3)	
Adjuvant Chemotherapy			<0.001
No	998 (19.20	187 (13.7)	
Yes	4191 (80.8)	1182 (86.3)	
Tumor location			0.019
colon	2289 (44.2)	653 (47.8)	
Rectum	2887 (55.8)	714 (52.2)	
Albumin (g l^−1^)			<0.001
<35	239 (4.7)	147 (11.2)	
≥35	4805 (95.3)	1167 (88.8)	
CRP (mg l^−1^)			<0.001
≤ 10	4776 (94.7)	1162 (88.4)	
>10	268 (5.3)	152 (11.6)	
GPS			<0.001
0	4776 (94.7)	1163 (88.5)	
1	139 (2.8)	46 (3.5)	
2	129 (2.6)	105 (8.0)	

*CRP, C-reactive protein; GPS, Glasgow prognostic score*.

### Univariate and multivariate regression analysis

Uni-variate Cox regression analysis determined clinical characteristics significantly associated with OS (Table 2) including leukocytosis, T- stage, N-stage, distant metastasis status, age, grade, lymph node examined(>12), mucinous adenocarcinoma, Perineural/Vascular invasion, adjuvant therapy and tumor location. Leukocytosis were also confirmed in the multivariate analysis as the independent prognostic factor for OS. Further analysis was conducted to investigate the benefit for DFS (Table 3), then univariate analysis found that leukocytosis, T- stage, N-stage, distant metastasis status, age, grade, lymph node examined(>12), perineural/vascular invasion, adjuvant therapy and tumor location were significantly associated with DFS. Multivariate analysis balancing those factors also showed that leukocytosis (HR = 1.146, *P* = 0.04) was an independent predictor of DFS.

**Table 2:** Univariate and multivariate analysis for overall survival.

	**Univariate analysis**			**Multivariate analysis**		
**Variables**	**Hazard ratio**	**95% CI**	***P*-value**	**Hazard ratio**	**95% CI**	***P*-value**
Leukocytosis	1.502	1.277–1.766	<0.001	1.341	1.133–1.587	0.01
Male gender	0.949	0.82–1.098	0.479	NA		
Age >70	1.811	1.541–2.128	<0.001	2.438	2.051–2.897	<0.001
T stage, T4	1.746	1.556–1.959	<0.001	1.352	1.103–1.657	0.004
N stage, N2	2.18	1.998–2.379	<0.001	1.48	1.321–1.657	<0.001
M1 stage	6.553	5.603–7.664	<0.001	4.952	4.173–5.875	<0.001
Grade(poor)	2.284	1.968–2.652	<0.001	1.423	1.207–1.677	<0.001
Lymph node examined (>12)	0.782	0.670–0.913	0.02	0.752	0.639–0.884	0.001
Mucinous Adenocarcinoma	1.624	1.269–2.078	<0.001	1.239	0.986–1.557	0.066
Perineural invasion	2.502	2.150–2.911	<0.001	1.373	1.162–1.622	<0.001
Vascular invasion	3.119	2.696–3.609	<0.001	1.628	1.370–1.935	<0.001
Adjuvant therapy	4.114	3.013–5.618	<0.001	0.833	0.480–1.445	0.515
Rectal cancer	0.763	0.660–0.882	<0.001	0.9	0.770–1.050	0.181
GPS (2)	1.491	1.305–1.703	<0.001	1.332	1.156–1.534	<0.001

**Table 3:** Univariate and Multivariate analyzes of prognostic factors for disease-free survival.

	**Univariate analysis**			**Multivariate analysis**		
**Variables**	**Hazard ratio**	**95% CI**	***P*-value**	**Hazard ratio**	**95% CI**	***P*-value**
Leukocytosis	1.284	1.131–1.458	<0.001	1.146	1.006–1.305	0.04
Male gender	1.035	0.927–1.156	0541	NA		
Age >70	1.325	1.132–1.552	<0.001	0.965	0.818–1.138	0.668
T stage, T4	1.752	1.607–1.909	<0.001	1.76	1.021–3.033	0.042
N stage, N2	2.04	1.912–2.177	<0.001	1.348	1.241–1.465	<0.001
M1 stage	8.157	7.304–9.108	<0.001	5.656	5.002–6.397	<0.001
Grade, poor	1.168	0.788–1.730	<0.001	1.064	0.941–1.203	0.323
LN examined >12	0.849	0.755–0.955	0.006	0.803	0.710–0.908	<0.001
Mucinous Adenocarcinoma	1.138	0.920–1.409	0.233	NA		
Perineural invasion	2.426	2.163–2.722	<0.001	1.316	1.161–1.492	<0.001
Vascular invasion	2.308	2.065–2.579	<0.001	1.151	1.010–1.311	0.035
Adjuvant therapy	5.582	4.280–7.280	<0.001	1.97	1.335–2.907	0.001
Rectal cancer	0.847	0.767–0.935	0.001	1.049	0.944–1.165	0.376
GPS (2)	1.18	1.048–1.327	0.006	1.128	1.0–1.273	0.05

### Extension of the relapse prognostic model with leukocytosis

Figure 3B2 illustrates the differences for tumor relapse (local or distant failure) in patients with or without leukocytosis. We could observe the patients with events in baseline leukocytosis were significant more compared to those without leukocytosis (*p* = 0.001). To improve the prognostic accuracy for current TNM staging system, we established a predictive model for colorectal cancers by combining TNM staging system and leukocytosis. The combination of both factors achieved the highest area under the curve (AUC) value (0.718 and 0.781), while AUC predicting OS and DFS based on the only TNM staging system was 0.711 and 0.779, see Figures 3C1,C2,D1,D2.

### Association between postoperative chemotherapy and leukocytosis

**Figure 4:**
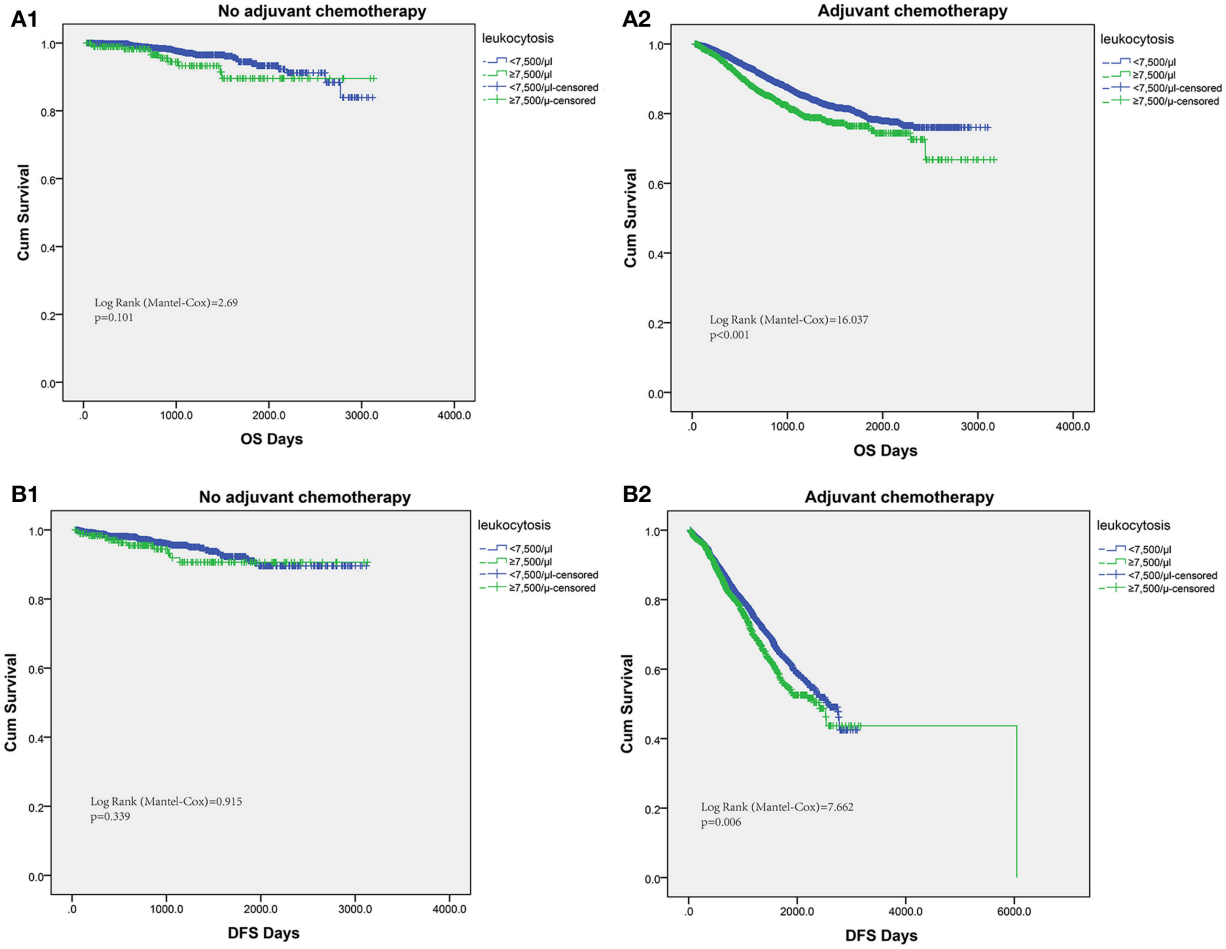
Subgroup analysis to assess predictive value of leukocytosis for chemotherapy benefit. Kaplan–Meier analysis of OS **(A1,A2)** and DFS **(B1,B2)** based on the leukocytosis status from patients with or without chemotherapy.

The host inflammation status was reported to contribute significantly to chemotherapy efficacy. Thus, we tried to assess the efficacy of fluorouracil-based chemotherapy according to the leukocytosis status in patients receiving chemotherapy. For patients without postoperative adjuvant chemotherapy treatment, the leukocytosis status was not significantly associated with OS or DFS. However, patients with leukocytosis had significantly shorter OS in patients with adjuvant chemotherapy (log-rank test = 16.037, *P* < 0.001). Similarly, the association between leukocytosis and DFS was also significant in patients with adjuvant chemotherapy (log-rank test = 7.662, *P* = 0.006, Figure 4).

### Development and validation of nomograms for predicting prognosis with leukocytosis

**Figure 5:**
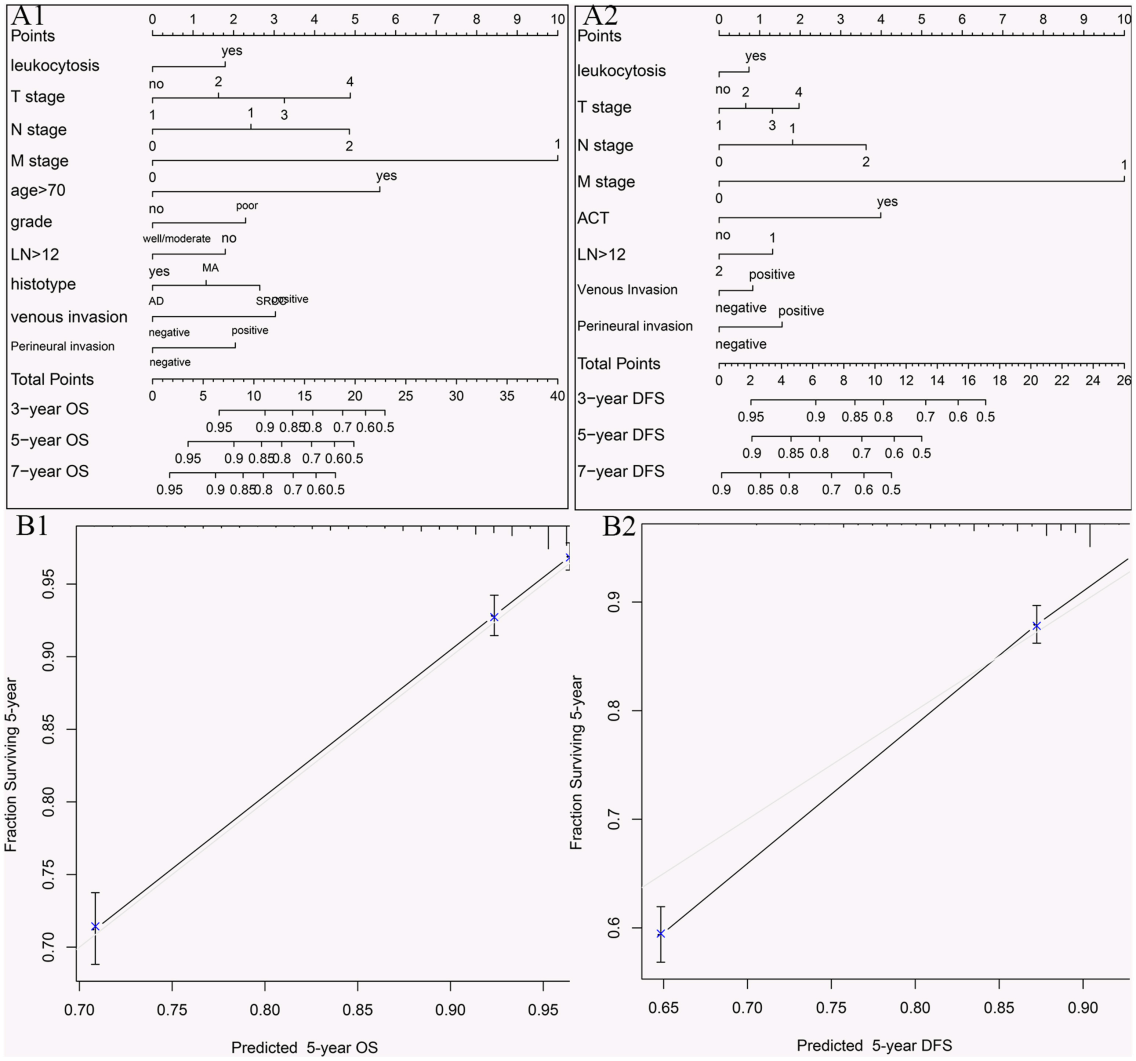
Nomograms for OS **(A1)** and DFS **(A2)** based on leukocytosis status and other well-recognized prognosticators. The calibration of nomograms for both OS **(B1)** and DFS **(B2)**.

To visualize the prognostic application of leukocytosis status, we then created nomograms for OS and DFS based on leukocytosis status and other well-recognized prognosticators (Figures 5A1,A2, respectively). Figures 5B1,B2 showed the calibration of nomograms for both OS and DFS, demonstrating a great prediction accuracy of this nomogram. In another word, calibration curves for two nomograms revealed no deviations from the reference line.

### Leukocytosis and intra-tumoral immune infiltration

**Figure 6:**
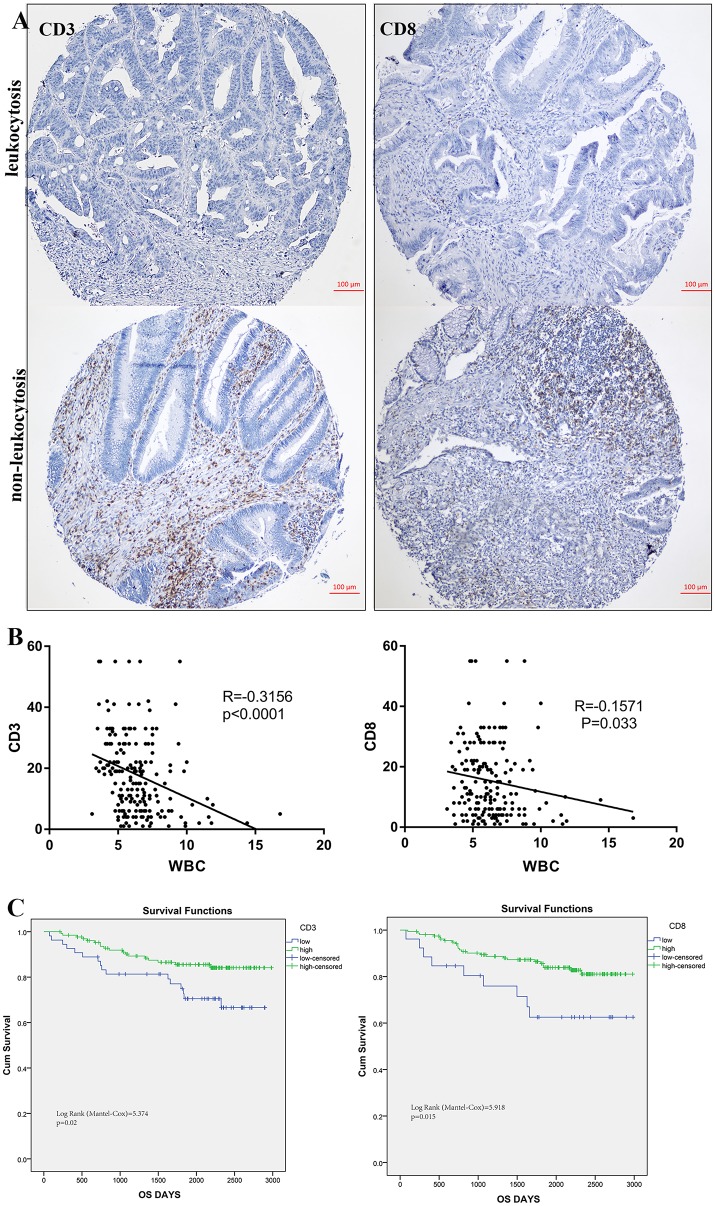
Leukocytosis and intra-tumoral immune infiltration. **(A)** representative immunohistochemical images showing high and low CD3+, CD8+ TIL in CRC with leukocytosis. **(B)** Association of peripheral WBC count with CD3+ and CD8+ T-cells infiltration. **(C)** Prognostic impact of CD3+ and CD8+ T-cells infiltration on survivals.

To illustrate the correlation of tumor-infiltrating inflammatory and immune cells with pre-treatment leukocyte count, we constructed tissue microarray based on leukocytosis status in 184 colorectal patients. Regarding the correlation of leukocyte count and immune cells infiltration, patients with pretreatment leukocytosis had a significantly lower intra-tumor CD3+ and CD8+ TIL infiltration (*p* < 0.001 and *p* = 0.033, Figure 6B) compared to patients with no leukocytosis. Representative images of low and high intra-tumoral CD3+ and CD8+ TIL were shown in Figure 6A. But we cannot find significant correlation between leukocyte and CD45+ and tumor-associated neutrophils (*p* > 0.05). In addition, Kaplan-Meier analysis showed that low intra-tumor CD3+ and CD8+ TIL infiltration were associated with worse prognosis of CRC patients (*P* = 0.02 and *p* = 0.015, Figure 6C).

## Discussion

In this current study, we have investigated the prognostic impact of peripheral blood leukocyte in the context of intra-tumoral immune profile. We validated leukocytosis as a prognostic factor predicting survivals and tumor response to adjuvant chemotherapy in patients with colorectal cancer. Leukocytosis was independently associated with OS and DFS among disease subgroups and across disease stages in this study. Our data confirmed these findings not only utilizing the established cutoff value of 10,000/μl as leukocytosis, but we also observed worse OS and DFS in patients with moderately elevated leukocyte, based on cutoff value of 7,500/μl. Hence, leukocytosis defined as the leukocyte count over 7,500/μl was identified for further analysis.

Inflammatory response has been associated with malignant lesions in several system, including head, neck, lung, and uterine/cervix. Even more, chronic inflammation was supposed to lead to the development of various malignant lesions and to the increasing risk of relapse (12). So in line with our results, baseline leukocytosis displayed a worse prognosis in oropharyngeal cancer (7), anal carcinoma (8, 13) and cervical cancer (9). Neutrophils are the most majority of peripheral leukocyte and are the first reaction responding to sites of inflammation and progression. And it was indicated that elevated levels of these intra-tumor leukocytes were correlated with poor prognosis through inducing metastasis by fueling the metastatic niche (14). Neutrophils infiltrating tumor tissues played essential roles in tumor development and progression (15). In fact, at the early stage of colorectal tumorigenesis, infiltration of neutrophils has been involved, as neutrophils infiltrated in colorectal adenomas much more than in adjacent normal mucosa and the count of neutrophils was positively correlated with adenoma size (16). In mechanically, neutrophils producing N-nitrosamines may be responsible, as they are known to be carcinogenic (17, 18). In addition, an hMSH2-dependent G2/M checkpoint arrest could be induced by activated neutrophils, then replication errors occurred in colon epithelial cells (19). These findings are consistent with our observations, indicating leukocytes as an adverse prognostic factor in cancer progression. In addition, inflammation leading to immunosuppression provided a preferred niche for tumorigenesis (20). Besides tumor-extrinsic inflammation caused by bacterial and viral infections, tumor-intrinsic inflammation triggered by cancer mutations all contributed to malignant transition through the recruitment and activation of inflammatory cytokines. Key mediators of inflammation-induced cytokines included NF-kappa B, STAT3 pathways, reactive oxygen/nitrogen species and prostaglandins (21). So in both our “training set” and “testing set,” leukocytosis independently decreased OS and PFS.

In this study, we found that tumor associated leukocytosis had a significant association with low intra-tumoral CD3+ and CD8+ TIL infiltration and vice versa. Several mechanisms and different lymphocytes subtypes have been indicated in the survival beneficial or detrimental effect of immune cells within human colorectal tumors (22–24). Leukocytes have been considered as one of first-line defenders against infectious microorganisms in acute inflammation, but in a chronically activated state, the persistent producing growth factors and reactive oxygen could result in permanent genomic alterations and impede lymphocytes recruitment by interacting with the DNA of the proliferating epithelium (25). Above all, leukocytosis can result to inhibit CD8+ TIL activation via upregulation of programmed death 1 (PD1) on T immune cells and myeloid cells, which is consistent with our findings (26). To the best of our knowledge, we have reported this phenomenon firstly and it proposes a possible explanation for the adverse prognosis of leukocytosis.

Furthermore, to identify more clinical implications of tumor associated leukocytosis additional of its survival prognosis, we also carefully evaluated the correlation between leukocytosis and chemotherapy. We observed that in patients with post-operative chemotherapy, those without leukocytosis were easier to have longer OS and DFS, compared with those with leukocytosis, while there was no correlation in patients without chemotherapy for leukocytosis. It implied that leukocytosis could be an important indicator for predicting the efficiency of chemotherapy. In line with our observation, inflammatory, and immune cells were showed be accounting for the efficacy of chemotherapy. As in hepatic cancer, CCL2 and CCL17 expressed from sustained inflammation could recruit macrophages and Treg cells promoting neovascularization and resistance to anti-angiogenesis therapy (27). A proportion of patients who do not benefit from chemotherapy are associated with a worse prognosis. In our population, they were characterized by baseline tumor associated leukocytosis protecting them from benefit from chemotherapy. Do these patients need treatment intensification? The association of leukocytosis with efficacy of chemotherapy might open doors for several novel therapeutic approaches in these patients by impeding the access of tumor-associated leukocytosis into growing tumors. Just as inhibition of neutrophil infiltration utilizing interleukin-8 antagonist was displayed to inhibit tumor growth, metastasis and angiogenesis of melanoma and lung cancer (28, 29). Hence, immune-monitoring of inflammation marker in blood and tumor tissue in colorectal patients seems essential.

The strength of this study is the validated association between tumor associated leukocytosis and poor survival as well as resistance to chemotherapy in this large cohort of colorectal cancers. The conventional clinical characteristics to predict treatment outcome in colorectal cancer are relatively inadequate. The addition of biological parameters to tumor associated leukocytosis might help us to precisely tailor therapeutic strategies for each personized patient. In this study, the addition of baseline tumor associated leukocytosis seems to improve the stratification of colorectal cancers into distinct groups based on the risk of treatment resistance. At the meanwhile, our study has several limitations. Firstly, the retrospective design could have led to selection bias. In second, although we identified the impact of the interaction of leukocytosis and T immune cells involved in tumor progression, the underlying mechanisms through which those major immune cells crosstalk with each other remains unrevealed. Thirdly, combined prognostic serum marker will be more precision to use in bedside, which are our next concern and under investigation.

## Conclusions

To date, this is the first study addressing the negative impact of pretreatment leukocytosis on survivals of patients with colorectal cancers. The worse survivals in patients with leukocytosis was mainly attributed by relapse and resistance to chemotherapy. In addition, the inverse correlation between leukocytosis and intra-tumoral CD3 as well as CD8+ TIL provided vital mechanistic insight on the adverse impact of peripheral leukocytosis. At last, the addition of leukocytosis to the conventional established prognostic indicators should help us to better tailor therapeutic strategies for each individualized patient.

## Ethics statement

This study was carried out in accordance with the recommendations of the ethics committee of FUSCC and approval from the institutional review board. All patients gave written informed consent in accordance with the Declaration of Helsinki.

## Author contributions

XH, J-JP, and S-JC conceived and designed the experiments. XH, Y-QL, S-JC, and Q-GL analyzed the data. XH and Y-LM contributed reagents, materials, and analysis tools. XH wrote the paper.

### Conflict of interest statement

The authors declare that the research was conducted in the absence of any commercial or financial relationships that could be construed as a potential conflict of interest.
